# Bony Regrowth and New Spur Formation as Possible Causes of Failed Distal Clavicle Excision—Mid-Term Outcomes after Revision Surgery in a Matched-Pair Analysis

**DOI:** 10.3390/jpm13081221

**Published:** 2023-08-01

**Authors:** Roman C. Ostermann, Martin Eigenschink, Philipp R. Heuberer, Paul Siegert, Willi Muellbacher, Lisa Anderl, Beate Schrott, Brenda Laky, Leo Pauzenberger, Werner Anderl

**Affiliations:** 1 Orthopedic Department, Vienna International Shoulder and Joint Clinic, 1030 Vienna, Austria; 2 2nd Orthopedic Department, Hospital of the Sacred Heart of Jesus, Baumgasse 20A, 1030 Vienna, Austria; 3 Austrian Research Group for Regenerative and Orthopedic Medicine (AURROM), 1030 Vienna, Austria; ordination@ortho-eigenschink.at (M.E.);; 4 AUVA Traumacenter Vienna, Meidling, 1030 Vienna, Austria; 5 1st Orthopaedic Department, Orthopaedic Hospital Speising, 1030 Vienna, Austria; 6 TUM School of Medicine, Technical University of Munich, 80333 München, Germany; 7 Center of Clinical Research (CCR), University Clinic of Dentistry, Medical University of Vienna, 1030 Vienna, Austria

**Keywords:** acromio-clavicular, AC-joint, revision, arthroscopy

## Abstract

Background: Despite high rates of successful outcomes after open and arthroscopic distal clavicle excision (DCE) for symptomatic acromioclavicular joint (ACJ) degeneration, some patients present with persistent symptoms and disabilities after surgical intervention. This study aims to compare radiological, functional, and subjective outcomes of open revision surgery after failed arthroscopic DCE to primary successful arthroscopic DCE. Methods: In this retrospective case-control study, 10 patients who underwent open DCE revision were age- and gender-matched with 10 patients who did not require revision surgery after DCE. Radiographic evaluation included presence of acromioclavicular spurs and acromioclavicular joint distance. Functional and subjective outcomes were assessed using the CS, SSV, SST, VAS for pain, patient’s satisfaction, ASES and quick DASH score. Results: At the latest postoperative follow-up (case: 57.3 ± 19.2 months; control: 63.5 ± 16.3 months), spur formation was detected in twice as many cases in the revision group, while acromioclavicular distance showed no significant difference. However, a significant bony regrowth was noticed in the revision group between revision surgery and latest follow-up, with a decrease of the acromioclavicular distance from 9.2 ± 1.6 mm to 5.9 ± 4.6 mm (*p* = 0.026) and a development of new spur formations in 30% of cases. There were no significant differences in overall CS between the revision and control group (*p* = 0.174) at final follow-up, but the control group scored significantly higher in the CS subgroups pain (*p* = 0.012) and internal rotation (*p* = 0.016). Mean SSV was significantly lower in the revision (65.5 ± 22.3%) compared to the control group (85.9 ± 16.4%; *p* = 0.031). Conclusions: Bony regrowth at the distal clavicle presenting as postoperative AC-distance narrowing and new spur formation was observed more distinctly in the revision group. Despite a slight increase in postoperative outcomes after revision surgery, subjective satisfaction and recalcitrant pain remain a concern. Level of Evidence: Therapeutic Level III, retrospective case-control study

## 1. Introduction:

Distal clavicle excision (DCE) is a common surgical procedure for failed non-operative treatment of symptomatic acromioclavicular joint (ACJ) arthritis or osteolysis [1,2,3]. In the last decade, a transition from open to arthroscopic DCE was noticed, which might be attributed to the more invasive nature and associated complications of the open technique [4]. The increasing popularity of arthroscopic DCE was also linked to the advantage of intraoperative diagnosis and treatment of concomitant intra-articular or subacromial shoulder disorders (e.g., rotator cuff tears, long head of biceps tendon pathologies, impingement), with reported incident rates of up to 50 percent [5]. However, open distal clavicle excision has remained ingrained as a surgical approach since first described in 1941, with the advantage of better visualization of posterosuperior parts of the ACJ and the possibility of open capsular plication for stability and open denervation [6,7].

However, both approaches aim to remove the entire degenerative tissue of the affected joint, including the articular disc, degenerated articular cartilage, and adjacent bone of the lateral clavicle and medial acromion, in order to expand the intra-articular space and avoid painful contact between the articulating bones, while preserving stabilizing structures [8].

While both arthroscopic and open DCE show overall good to excellent clinical results in 76–100 percent of the cases, adequate long-term pain relief cannot be achieved in all cases, with some patients presenting with recalcitrant complaints after index surgery [1,9]. Surgical failure has been attributed to both under and over- resection, postoperative joint instability, stiffness, heterotopic ossification and spur formation, or under-diagnosed concomitant pathologies [9,10,11,12]. After a thorough reevaluation, revision surgery might be indicated [13]. To this date, there is a lack of clinical results following revision DCE in the literature.

This study aims to compare radiological, functional, and subjective outcomes of open revision surgery after failed arthroscopic DCE to primary successful arthroscopic DCE. We hypothesized that patients requiring revision surgery are more likely to present with persistent pain due to either decreased acromioclavicular distance or spur formation after primary DCE compared to patients with successful DCE.

## 2. Methods

### 2.1. Study Population

This retrospective study matched two independent cohorts for analysis. The institutional ethics committee approved this study prior to investigations. All surgical procedures were performed by fellowship-trained shoulder surgeons.

For the first cohort (group A), all patients who underwent open revision surgery within five years after primary arthroscopic DCE between January 2005 and December 2009 at a single institution were identified. Inclusion criteria were (1) male or female ≥ 18 years of age, (2) positive cross-body adduction test, (3) isolated ACJ complaints (persistent ACJ pain and/or clinically relevant ACJ instability), and (4) minimum follow-up of two years. All patients of this group had positive lidocaine infiltration test to the ACJ. Exclusion criteria were previous shoulder surgeries besides the index DCE surgery and secondary diagnosed concomitant glenohumeral or subacromial pathologies during revision surgery. Ten patients (three males and seven females) aged between 41 and 71 years at time of index surgery qualified for inclusion into group A.

The second cohort (group B) consisted of patients who underwent primary arthroscopic DCE between January 2005 and December 2009 at the same institution, without revision surgery. Inclusion criteria were the same as for group A. Patients with persistent ACJ complaints at final follow-up and any revision surgery were excluded. Group B was matched to group A regarding age, gender, and concomitant pathologies at index surgery.

Patient-specific parameters, including surgery-related data such as involvement of dominant shoulder, operation time (minutes), and length of hospital stay (days), were recorded for both groups.

### 2.2. Surgical Techniques

In the arthroscopic approach, the patient was placed in a beach-chair position. Two standard arthroscopic portals were established. After arthroscopic inspection of the glenohumeral joint for concomitant shoulder pathologies, the arthroscope was inserted into the subacromial space. The acromioclavicular joint was located by inserting a spinal needle from cranial through the joint into the subacromial space, followed by an incision of the inferior articular capsule, while carefully protecting the posterior and superior portion of the capsule through an anterior portal. A resection of the lateral clavicle and adjacent acromion with a 4 mm burr was performed. The resection was considered sufficient once the burr could easily fit between the articulating bony structures. Care was taken not to resect more than 8 mm to leave the superior joint capsule and CC ligaments attached to the lateral clavicle.

As with the arthroscopic technique, in the open approach for revision cases, the patient was positioned in a beach-chair position. A 2 to 3 cm skin incision was performed from anterior to posterior right above the AC joint. The fascia was incised perpendicular to the AC joint to expose the articular capsule. After splitting the capsule, the periosteum was elevated and the resection was carried out using an osteotome, oscillating saw, burr, or rongeur. The lateral clavicle was resected in a slight angle from superomedial to inferolateral, to parallel the normal orientation of the joint for a better congruency with the acromion. Resection length was 3–4 mm of the lateral clavicle and 2–3 mm of the adjacent acromion. By performing a flexion and adduction of the arm, the joint was examined for possible residual contact of the two articulating bones during movement. Any inferior bony spurs were additionally resected. Care was taken to perform a thorough suture of the capsule and delto-trapezial fascia, as it determines the stability of the joint postoperative. Non-absorbable sutures were used to reach watertight closure of the capsule and the delto-trapezial fascia.

### 2.3. Clinical and Radiographic Assessment

A comprehensive physical evaluation, including measurement of active abduction, forward flexion, and external and internal rotation using a goniometer, was conducted by two shoulder fellows (LA, BS). A cross-body adduction test for ACJ complaints was performed [3]. The Constant score (CS) [14] (0–100 points; 100 presenting the best possible outcome) was obtained as the primary clinical outcome measure. For subjective measures, visual analog scale (VAS) [15] for pain (0–10 points), Subjective Shoulder Value (SSV; 0–100%; 100% presenting the best possible outcome) [16], Simple Shoulder Test (SST) [17], patient’s satisfaction (very satisfied, satisfied, fairly satisfied, or not satisfied), American Shoulder and Elbow Surgeons (ASES) [18] score (0–100 points; 100 presenting the best possible outcome), and the Quick Disabilities of the Arm, Shoulder, and Hand (QuickDASH) [19] score (0–100 points; 100 presenting the best possible outcome) were assessed.

**Figure 1:**
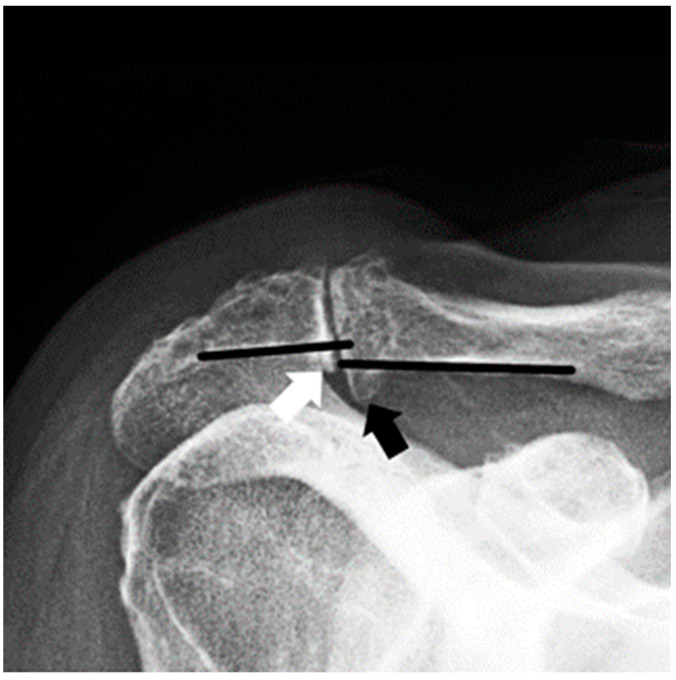
**Zanca view radiograph of a right shoulder.** The black arrow shows an inferior bony spur of the distal clavicle, the white arrow shows a small inferior bony spur of the medial acromion.

All patient received plain radiographic imaging, including an anteroposterior, axial and Y-view, and a Zanca view, pre- and immediately postoperative (=perioperative) as well as at the latest follow-up. Images were evaluated for acromioclavicular spur formation, and acromioclavicular joint distance (ACD; mm) was measured on Zanca views. A spur was defined as a definite bone formation inferior to the distal cortex of the lateral clavicle or the medial acromion (Figure 1). ACD and spur evaluations were carried out by two independent shoulder surgeons (PRH, LP) [20]. Additionally, postoperative computed tomographic (CT) scans (day one following surgery) of the affected AC joints were obtained for patients undergoing revision surgery (group A).

### 2.4. Statistical Analysis 

Patient details were presented using descriptive statistics. Quantitative data were expressed as means with standard deviation or median and range. For continuous and normal distributed data, the Student’s *t*-test was applied and the Mann–Whitney U test was used for ordinal or non-normally distributed data to determine differences between two independent groups. A Pearson Chi-square or Fisher’s exact test was performed to analyze categorical variables. Paired *t*-tests (parametric) and Wilcoxon Signed-Rank tests (non-parametric) were performed to compare between time points within a group. All comparative tests were two-tailed and statistical significance level was set at *p* < 0.05. Statistical analyses were performed using IBM SPSS Statistics 24.0 software (IBM^®^ Corporation, Armonk, NY, USA).

## 3. Results

In group A, one patient presented without any additional pathologies regarding the shoulder before the index surgery, whereas the other nine patients presented with additional shoulder-related comorbidities, including subacromial impingement (*n* = 8), rotator cuff lesions (*n* = 4), and long biceps tendon pathology (*n* = 2). All concomitant shoulder pathologies were treated surgically during index arthroscopic surgery with antero-lateral subacromial decompression, rotator cuff repair, and LHBT tenotomy, respectively. The average surgery time from incision to suture of the index surgery in this group was 48.3 ± 8.5 min, and the average length of hospital stay for the primary ACR was 2.2 ± 1.0 days. Revision surgery was indicated after a mean of 10.0 ± 8.4 months following the index surgery for persistent pain. The latest follow-up was performed 57.3 ± 19.2 months after index surgery.

In group B, one patient presented without any additional pathologies. The other nine patients presented with subacromial impingement (*n* = 8), rotator cuff lesions (*n* = 4), and long biceps tendon pathologies (*n* = 2), which were all treated surgically during the index procedure similar to group A. The average surgery time from incision to suture in group B was 35.3 ± 12.6 min, and the average length of hospital stay was 2.9 ± 1.1 days. The mean follow-up time was 63.5 ± 16.3 months.

A comparison between the age- and gender-matched cohorts showed no significant differences regarding demographic data (Table 1).

### 3.1. Radiographic Assessment

**Figure 2:**
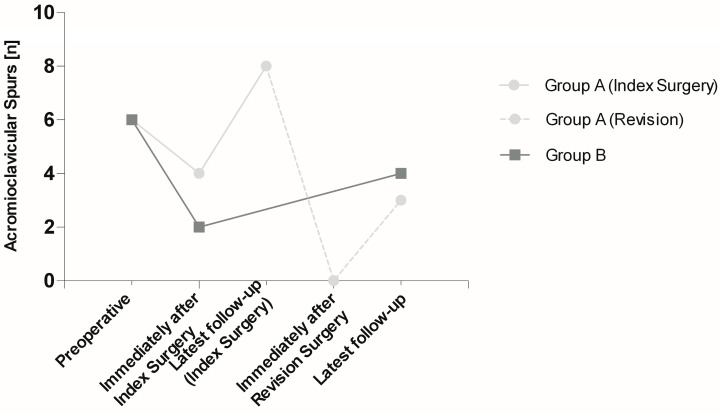
Detection of acromioclavicular spurs in absolute numbers (n) upon radiographic imaging. Group A (10 patients) was evaluated preoperatively, immediately after index surgery, at latest follow-up before revision surgery, immediately after revision surgery, and at latest follow-up, while group B (10 patients) was evaluated preoperatively, immediately after index surgery, and at latest follow-up.

Before index surgery, six patients in group A showed inferior acromioclavicular spurs. According to perioperative X-rays, one day after index surgery, spurs were detected in four patients. The X-rays directly before revision surgery showed that the number of patients with spurs increased to eight. All spurs were resected during revision surgery. Thus, no spurs were detected on postoperative X-rays and CT scans immediately following revision surgery. At latest follow-up of group A, three patients presented with spurs again. In group B, a decrease of spurs from six patients preoperatively to two patients directly after index surgery was noticed. The number of patients with spurs in group B increased to four at the latest follow-up. Comparing the spur formation before revision surgery in group A, which is the latest follow-up of index surgery in this group, and the latest follow-up in the control group B, twice as many patients with spurs were detected in group A (Figure 2).

**Figure 3:**
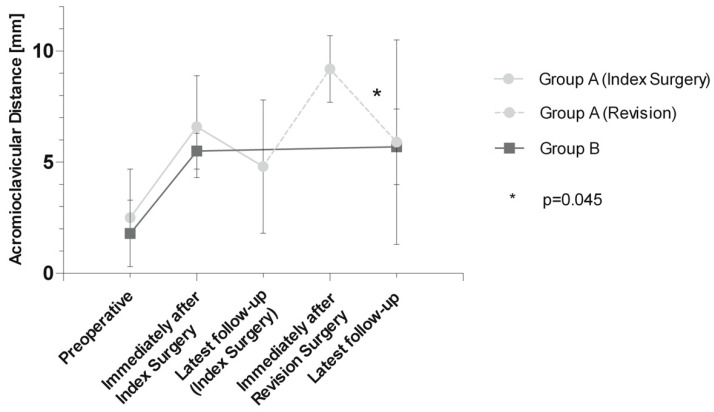
Acromioclavicular distance (ACD) in millimeters in both groups. Group A (10 patients) was evaluated preoperatively, immediately after index surgery, at latest follow-up before revision surgery, immediately after revision surgery, and at latest follow-up, while group B (10 patients) was evaluated preoperatively, immediately after index surgery, and at latest follow-up. Significant *p*-values are indicated with *.

The acromioclavicular distance in group A was 2.5 ± 2.2 mm before and 6.6 ± 2.3 mm immediately after index surgery, and decreased to 4.8 ± 3.1 mm before revision surgery. In group B, the acromioclavicular distance changed from 1.8 ± 1.6 mm before to 5.5 ± 0.9 mm immediately after surgery and remained stable with 5.7 ± 1.7 mm at latest follow-up (Figure 3).

While the decrease in acromioclavicular distance from peri- to the next postoperative follow-up regarding index surgeries of both groups was not significant (group A: *p* = 0.168; group B: *p* = 0.751), acromioclavicular distance significantly decreased from peri- (9.2 ± 1.5 mm) to the last follow-up (5.9 ± 4.6 mm; *p* = 0.045) regarding the revision surgery of group A.

### 3.2. Functional and Subjective Assessment

A comparison between group A and B of all functional and subjective outcome measures at the latest follow-up showed, with the exception of CS pain, internal rotation, and SSV, no significant differences (Table 2).

Similar overall CS and CS subgroups of group A before revision surgery compared to the latest follow-up were detected (Table 3).

ADL, activity of daily living; ROM, range of motion; CS, Constant Score; qDASH, Quick Disabilities of the Arm, Shoulder, and Hand score; ASES American Shoulder and Elbow Surgeons score; SSV, Subjective Shoulder Value; SST, Simple Shoulder Test; VAS, visual analog scale.

The majority of patients were very satisfied (group A: 60%; group B: 60%) or satisfied (group B: 40%) with the procedure. The remaining patients in group A were fairly satisfied (30%), while one patient was not satisfied.

## 4. Discussion

The most important finding of the current study was that, although a slight increase in postoperative clinical outcome could be achieved with surgical revision after failed DCE, subjective satisfaction and recalcitrant pain remain a concern. While resection length appeared to have no influence on outcomes, increased spur formation in failed DCE was noticed. Furthermore, bony regrowth at the distal clavicle resulting in AC-distance narrowing was observed and could be a possible cause for persistent pain and lower patient satisfaction following revision DCE.

To the best of our knowledge, this is the first study to report on radiological, functional, and subjective outcomes after revision distal clavicle excision. Firstly, we observed a bony regrowth at the distal clavicle representing a postoperative AC-distance narrowing and new spur formation, which could well be a possible cause for revisions. Secondly, patients with no revision after arthroscopic DCE seemed to have better internal rotation and reported slightly better subjective outcomes (CS pain, SSV, satisfaction with the procedure). Furthermore, only half of the patients seemed to be satisfied with their clinical outcomes following open revision DCE.

Even though high rates of successful outcomes have been reported for both open and arthroscopic primary DCE, treatment failures occur, and some patients remain with persistent symptoms after DCE [1,12,13]. Those patients remain challenging, and treatment options are limited. This study pursued the objective to compare clinical and subjective outcomes in patients who required revision surgery after arthroscopic DCE to patients that did not require further treatment after index DCE, to gain insight into factors for a successful treatment. Furthermore, we aimed to determine the effect of open revision surgery on the patient’s satisfaction.

While the overall clinical outcome in both groups did not differ significantly, one of the main findings in our study is that the subjective outcome was significantly worse in the revision group. Our data showed that, although the active range of motion and activities of daily living were only slightly inferior to the control group, persistent ACJ pain leads to inferior subjective outcome measures. In this study, only internal rotation was impaired in the revision group, which could be attributed to pain that arises due to rotation in the ACJ during maximum glenohumeral internal rotation, rather than musculoskeletal limitation. Although we observed an overall trend of increased Constant score from before to after revision surgery, the difference was marginal and not statistically significant. Ultimately, only a little over half the patients were satisfied with open revision surgery, while the rest were not. In accordance with our findings, Kharrazi et al. found 45% of patients with ongoing symptoms and continued pain following revision DCE in their study [13].

The ideal extent of resection in DCE has been reported as a key factor for successful treatment, but is still discussed controversially [3,12,21,22,23,24,25,26]. If the resection was inadequate, meaning there is still residual bone contact after surgery, the pain is likely to remain after the procedure. On the other hand, extensive resections can lead to disruption of the acromioclavicular and coracoclavicular ligaments and, consequently, to postoperative iatrogenic instability of the joint, which is equally associated with pain [27]. When we compare the measured resection length directly following index surgeries in both groups, we did not observe significant differences. Hence, at least in this small group, we could not identify resection length as the key factor for failed DCE. Eskola et al. [22] found a correlation between over-resection and postoperative pain. In our cohort, we could not find either significant positive nor negative correlations between the perioperative acromioclavicular distance and pain measurements. Therefore, the hypothesis was not confirmed. Interestingly, though, we observed a distance decrease of around 1.8 mm between the articulating bones from directly after index surgery to before revision surgery (10.0 ± 8.4 months), and a decrease again from around 3.3 mm at latest follow-up of revision surgery compared to the postoperative CT-scans directly after revision (46.9 ± 16.1 months). This finding could either be attributed to a regrowth of bone tissue around the resected joint or a tightening of capsular structures. A bony regrowth of the lateral clavicle has been reported before and identified as a possible source of reoccurring symptoms [9,28].

Another hypothesis was that patients requiring revision surgery are more likely to present with a postoperative prominent inferior acromioclavicular spur compared to patients without failed DCE. Comparing the two groups in regard to postoperative acromioclavicular spurs, we found no difference at latest follow-up. We made the observation, though, that in the revision group the number of patients presenting with acromioclavicular spurs increased from directly postoperative after the primary DCE to preoperative before the revision surgery from four patients to eight patients. In addition, we noticed four remaining spurs directly after index surgery. In those cases, the surgeons did not perform an additional coplaning of the distal clavicle, as the spurs did not seem to interfere with underlying structures intraoperatively. Those remaining spurs, however, could as well be a possible cause for the late revision. Nevertheless, after all spurs had been resected during revision surgery, another regrowth of spurs could be observed at latest follow-up following revision surgery in two patients. This finding seems to be consistent with the statement of Levine et al. [11], that the postoperative development of spurs can cause late failure of acromioclavicular joint resection and necessitate revision surgery. Interestingly, if we compare the number of patients presenting with spurs directly before revision surgery in group A with the latest follow-up in group B, we observe twice as many spurs in the revision group. Hence, spur formation could potentially contribute to the need of late revision, which could suggest a benefit of removing any spurs during the index surgery.

### Limitations

There are several limitations to this study. The current study is subject to the inherent limitations of a retrospective study design. As the number of patients undergoing revision surgery after DCE is low, the sample size of the current study is small. Although the study might be underpowered to reveal significant differences between groups, it provides novel data on a rare but clinically challenging condition. To counteract the limited cohort sizes, a matched cohort design and strictly defined inclusion criteria were employed for better comparability. Another potential bias is the difference in follow-up time. Furthermore, a limiting factor for this and other studies is the isolated evaluation of the AC joint. Pathologies affecting this joint are likely accompanied by other shoulder related comorbidities that could have an impact on the functional and subjective outcome. However, this study is one of very few studies, if not the first, reporting radiological, functional, and subjective outcomes after revision distal clavicle excision.

## 5. Conclusions

Bony regrowth at the distal clavicle presenting as postoperative AC-distance narrowing and new spur formation was observed more distinctly in the revision group. Resection length appeared to have no influence on outcome in our cohorts. Despite a slight increase in postoperative outcomes after index and revision surgery, subjective satisfaction and recalcitrant pain remain a concern in DCE revision surgery.

## Figures and Tables

**Table 1:** Comparison of demographic data between both groups at the time of primary acromioclavicular joint resection.

	Group A *n* = 10	Group B *n* = 10	*p*-Values
Age (years)	60.4 ± 8.9	60.8 ± 9.2	0.923
Gender (female/male)	7:3	7:3	1.000
Concomitant pathologies (yes/no)	9:1	9:1	1.000
Dominant arm involved (yes/no)	6:4	4:6	0.656

**Table 2:** Clinical and subjective outcome measures at latest follow-up of group A (revision) and group B (control). Respected measurements with standard deviations are indicated. Statistical differences are marked with *.

Scores	Group A *n* = 10	Group B *n* = 10	*p*-Values
Overall CS [points]	64.1 ± 19.3	74.9 ± 14.4	0.175
Pain	6.3 ± 6.0	13.0 ± 2.6	0.007 *
ADL	12.9 ± 5.7	17.0 ± 3.3	0.069
ROM	32.0 ± 6.0	34.6 ± 5.3	0.318
Abduction	8.4 ± 2.3	8.8 ± 2.5	0.714
Flexion	9.0 ± 1.7	9.0 ± 2.2	0.999
Internal rotation	5.0 ± 2.2	7.3 ± 1.0	0.012 *
External rotation	9.6 ± 1.3	9.6 ± 0.8	0.999
Strength	12.9 ± 6.9	10.3 ± 5.8	0.372
qDASH (points)	44.1 ± 31.3	24.3 ± 27.5	0.150
ASES (points)	56.7 ± 32.7	75.0 ± 24.3	0.172
SSV (%)	65.5 ± 22.3	85.9 ± 16.4	0.031 *
SST (points)	6.1 ± 3.6	7.9 ± 3.6	0.281
VAS (points)	4 (range 0–7)	1 (range 0–4)	0.123

**Table 3:** Overall and subgroups of Constant score in group A before revision surgery and after revision surgery at latest follow-up.

Constant Score (Points)	Group A Before Revision	Group A After Revision (latest FU)	*p*-Value
Pain	5 (range, 0–15)	5 (range, 0–15)	0.478
ADL	7.5 (range, 4–20)	10.5 (range, 6–20)	0.284
ROM	24 (range, 16–38)	34 (range, 20–38)	0.185
Strength	8 (range, 4–26)	13.5 (range, 1–22)	0.575
Overall CS	51.4 ± 22.1	64.1 ± 19.3	0.316

## Data Availability

Not applicable.

## List of Abbreviations

ACD	Acromioclavicular joint distance	DCE	Distal clavicle excision
ACJ	Acromioclavicular joint	qDASH	QuickDASH score
ASES	American Shoulder and Elbow Surgeons score	SST	Simple shoulder test
CS	Constant Score	SSV	Subjective Shoulder Value
CT	Computed tomography	VAS	Visual analog scale
